# Advantages of surgical simulation in the surgical reconstruction of oncological patients

**DOI:** 10.4317/medoral.22336

**Published:** 2018-09-28

**Authors:** Fernando Iglesias-Martín, Luis-Guillermo Oliveros-López, Ana Fernández-Olavarría, María-Ángeles Serrera-Figallo, Aída Gutiérrez-Corrales, Daniel Torres-Lagares, José-Luis Gutiérrez-Pérez

**Affiliations:** 1 DMD, MSc. Oral and Maxillofacial Surgeon. Virgen del Rocio Hospital; 2 DDS, MSc. Oral Surgeon. University of Seville; 3 PhD, DDS, MSc. Professor of Oral Surgery. Chairman of Oral Surgery. Department of Stomatology. University of Seville; 4 PhD, DMD, Professor of Oral Surgery. Chairman of Oral Surgery. Department of Stomatology. University of Seville

## Abstract

**Background:**

Stereolithography, which consists of computer-aided designed/computer-aided manufactured (CAD-CAM) and computer simulations, is a manufacturing technologies used for the production of definitive models and prototypes printed in three dimensions, and is widely used in Oral and Maxillofacial Surgery. Surgical procedures using models made by these technologies offer several advantages.

**Material and Methods:**

This article describes three clinical cases of our experiences with patients diagnosed with squamous cell carcinoma and mandibular osteosarcoma, who underwent surgical removal of the lesions and subsequent mandibular reconstruction with a free fibula graft using surgical guides.

**Results:**

In all three clinical cases, surgical guides were used for the mandibular osteotomy, fibula osteotomy, and graft placement in the recipient area.

**Conclusion:**

Surgical guidelines are useful for improving the accuracy of surgical interventions and are appropriate for many types of resection and mandibular reconstruction.

** Key words:**Fibula flap, virtual surgical planning, mandibular reconstruction, precise medicine.

## Introduction

Oral and Maxillofacial Surgery covers several areas considered sub-specialties, such as maxillofacial oncology, maxillofacial traumatology, orthognathic surgery, implantology, among others, in which the advancement of technologies with a combination of medicine and engineering have a very important role in the diagnosis and treatment of each patient in order to obtain a better postoperative and rehabilitation (1).

Mandibular reconstructions have been a great challenge for the oral and maxillofacial surgeon. The four basic principles for success of a reconstruction are to establish an ideal occlusal plane, a stable bone fixation, a good vascularization of the soft tissues at the time of covering the defect, and the vascularization of the graft after contact with the receiving area (2).

Traditionally, mandibular defects created by a resection of the area have been reconstructed using different donor sites, materials, and surgical techniques, including the free fibula flap. In order to obtain a reconstruction of the mandible, several osteotomies of the fibula fragment must be performed on similar characteristics of that fragment of the mandibular bone to be replaced (or as close as possible), providing a correct mandibular position and a good occlusion. Even so, traditional methods, regardless if the specialist has the necessary experience, are considered inexact and time-consuming surgical procedures (2-4).

The concept of computer-assisted surgery uses surgical simulation and three-dimensional tools (3D-CAD/CAM), such as cutting guides and insoles, rather than relying exclusively on the intraoperative manual approach (5).

Several studies published in the current literature show that computer-assisted surgical procedures, including surgical guides, increase their accuracy by offering several advantages.

Authors like Jeong *et al.* (3) have published about the importance of using surgical guides in osteotomies of the fibula in order to transfer the parameters of computerized surgeries to the real surgical scenario, noting that the fibula surgical guides allow a much more precise design of the osteotomy.

In 2016, Yuan *et al.* (4) concluded in a published study that surgical guides and digitally designed fixation plates were key in facilitating mandibular resection and reconstruction, and recommended their application in more clinical cases to demonstrate their reliability and importance.

Rustemeyer *et al.* (5) mentioned that the main advantage of computer-guided surgery is a smaller deviation between the reference bone points in the reconstructed mandibular area and the donor zone. They also pointed out that virtually planned surgery requires a shorter intervention time than conventional ones, resulting in a shorter ischaemia time, which are crucial to the success of craniofacial reconstruction microsurges, even with a greater number of necessary osteotomies.

Surgical guidelines are widely used in mandibular resections, yet these devices are considered difficult to design and manufacture (4). We present three clinical cases in which we will describe the importance of the splint or surgical guide in the free fibula graft for mandibular reconstructions that were observed and discussed with patients diagnosed with squamous cell carcinoma of the mandible and mandibular osteosarcoma.

## Clinical Cases

In the presented clinical cases, the Tumor Committee of the Department of Oral and Maxillofacial Surgery at the Hospital Virgen del Rocío was consulted for treatment approach and planning.

Before the surgery, a three-dimensional (3D) computerized tomography of the facial bones and fibula area was performed on each patient.

The donor virtual bone zone was designed and placed using the tools contained in the CAD-CAM planning software (VirSSPA Servicio Andaluz de Salud, Spain) and this procedure was carried out by a virtual computer simulation of the mandibular defect where the segmented fibula reconstructed the appropriate angulation in the original mandible. From this simulation, segments of a minimum of 2 cm were created and applied to the virtual mandibular defect. From these results, surgical guides of the osteotomy and free grafting of the fibula were obtained.

The osteotomy and graft-making guides designed for each patient were created using 3-D sterolithographic printers of the same planning software (VirSSPA Servicio Andaluz de Salud, Spain) in order to provide the amount of bone needed for the rehabilitation. For accurate reconstruction of the mandible, it was necessary to determine the correct angles between the multiple osteotomies. Therefore, we used our computer simulation results in a subsequent surgery to minimize bone spaces between the multiple bone segments.

All patients signed an informed consent for the publication of their medical history and photographic images of their evolution.

- Case 1

A 69-year-old female patient diagnosed with squamous cell carcinoma of the mandibular gingiva, a left retromolar trigone with mandibular involvement, and a metastatic cervical lymphadenopathy (stage IV (T4N2M0)). It was decided to perform an excision with segmental resection of the mandible plus functional cervical dissection and reconstruction with a microvascularized flap of the fibula. The surgery was previously planned using CAD/CAM technology.

**Figure 1:**
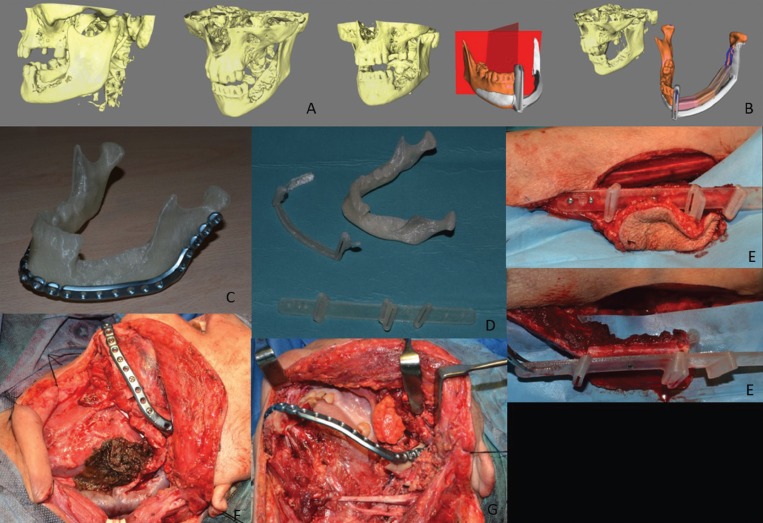
A - Virtual simulation of the initial mandibular defect. B - Virtual design of the mandibular osteotomy surgical guide. Virtual reconstruction of the mandibular defect with three segments of fibula graft. C - Design of the mandibular reconstruction plate on a stereolithographic model. D - Manufacture of the surgical guides of a mandibular osteotomy and a fibula graft. E - Intraoperative positioning of a surgical guide of the fibula for the grafting of the microvascularized graft. F - Placement of an osteosynthesis plaque for a mandibular reconstruction. G - Placement of reconstructive plate that were prepared for mandibular reconstruction.

In the first place, a virtual simulation of the initial mandibular defect was carried out, which allowed the design of a stereolithographic model, a mandibular osteotomy guide, and the creation of a reconstruction plaque on the model. From there, the virtual reconstruction of the mandibular defect was made with three segments of the fibula graft and the surgical guide was made to take the graft (Fig. 1A-1G).

The patient underwent radiotherapeutic and chemotherapeutic treatment on the tumor site, considering the postsurgical margins.

At 12 months of follow-up, after performing a FNAP in the right mandibular induration and right submandibular area, a recurrence of squamous cell carcinoma with signs of necrosis, treated with chemotherapy and palliative radiotherapy was diagnosed.

- Case 2

A 49-year-old patient diagnosed with epidermoid carcinoma in the left mandibular body and branch (stage IV (T4N1M0)). It was decided to perform a segmental tumor resection and reconstruction with a free fibula flap. Before the surgery, the design was made using CAD/CAM technology for the stereolithographic models, as well as the osteotomy and graft-taking guides.

**Figure 2:**
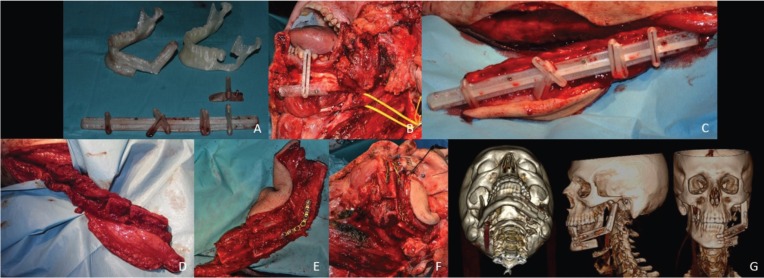
A - Design and manufacture of the surgical guides for the mandibular osteotomy and the fibula graft for the mandibular reconstruction. B - Positioning and intraoperative fixation of the surgical guide for the mandibular osteotomy. C - Positioning and intraoperative fixation of the surgical fibula osteotomy guide. D - Multiple osteotomies of 2-3 cm in the osteomyocutaneous fibula graft. E - Preparation and fixation of the segments, 2 to 3 cm, for transport and subsequent mandibular reconstruction. F - Mandibular reconstruction previously prepared with free fibula graft segments. G - Postoperative 3-D computed tomography of the mandibular reconstruction with a fibula graft.

The mandibular osteotomy was performed following the surgical guide previously made. Similarly, once the microvascularized graft of the fibula was exposed, the fibula osteotomy guide was positioned and fixed. Once the osteomyocutaneous graft was released, multiple osteotomies of 2-3 cm were required. For the transportation and subsequent mandibular reconstruction, the segments were fixed with titanium plates that were designed on the stereolithographic model (Fig. 2A-2G).

The patient underwent radical intention radiotherapy treatment after induction chemotherapy.

After 8 months of the surgical intervention, the patient suffers a relapse of infiltrating squamous cell carcinoma, moderately differentiated.

- Case 3

A 16-year-old patient diagnosed with mandibular osteosarcoma from two excisional biopsies, stage III (T2N0M0). After being assessed by the tumor committee, given the local extension, it was decided to initiate treatment with methotrexate at high doses and cisplatin-adriamycin with close monitoring of local evolution. After the end of the neoadjuvant chemotherapeutic treatment, a right hemimandibulectomy was decided by an intraoperative navigation to verify the limitation of the tumor recession and the reconstruction of the mandible with free fibula flap.

**Figure 3:**
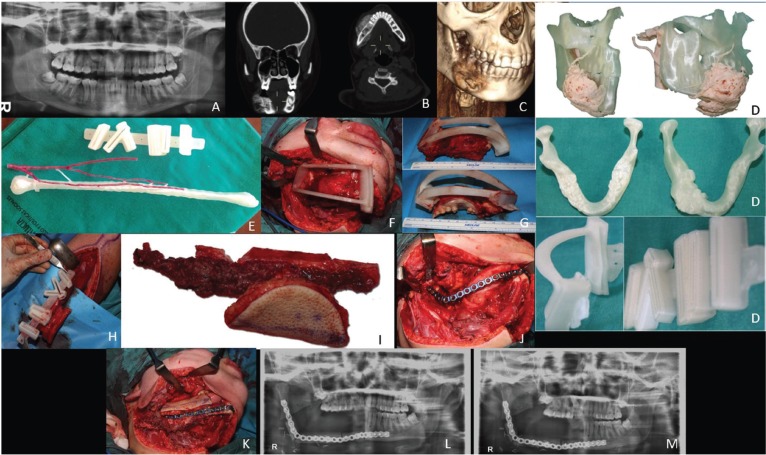
A - Preoperative orthopantomography. B - Coronal and transverse tomographic sections of the tumor lesion in the mandible. C - 3-D computed tomography of the tumor lesion. D - Manufactured surgical guides of the mandibular osteotomy and the fibula graft. E - A manufactured stereolithographic model of the fibula with its arterial vascularization and surgical guide for grafting. F - Positioning and intraoperative fixation of the surgical guide of the mandibular osteotomy. G - Surgical guide of the mandibular osteotomy after surgical resection of the tumoral lesion. H - Positioning and intraoperative fixation of the surgical fibula osteotomy guide. I - Multiple osteotomies of 2 to 3 cm in the osteomyocutaneous graft of the fibula. J - Positioning of the osteosynthesis plaque for the mandibular reconstruction. K - Mandibular reconstruction with free fibula graft segments. L - Postoperative orthopantomography two weeks after surgery. M - Postoperative orthopantomography six weeks after surgery.

Prior to the surgery, the CAD/CAM design was used to make stereolithographic models, both osteotomy and graft guides (Fig. 3A-3M).

After 12 months of the surgical intervention, the patient continues in follow-up, asymptomatic, showing significant improvement and radiographic stability.

## Discussion

Surgical computer-simulated procedures appeared first in the early 1990s (3). Despite the great advances in these technologies, the application of these virtual surgeries have limited scientific evidence. This is due to the complexity in the manipulation of the different software, the high costs of these technologies, and the difficulty in applying the results obtained during computer simulation in an actual surgery (3). Nevertheless, these drawbacks have been declining in recent years.

Several studies have demonstrated the importance of surgical guides for mandibular osteotomy and fibula osteotomy (3,5-8). Even so, they are considered difficult to design and manufacture by the surgeon (3,5,6).

Reconstruction of the mandibular defects requires complex surgeries, a high aesthetic and functional demand, and due to their particular anatomical characteristics, it is difficult to adequately shape the fibula once it is transferred from the donor site to the recipient site because the fibula has a rigid texture and requires additional fixing plates. These difficulties often lead to unsatisfactory results and cause many complications (4,9-11).

With the introduction of virtually-designed surgical guides, this complexity decreases, as these guidelines allow the surgeon to plan the reconstruction with greater precision to maximize the final result (4,12).

Several studies have pointed to a greater accuracy in the design of fibular graft osteotomies with the use of computer-aided surgical guides (3,4,12-19). In a control case study of 38 patients treated by mandibular reconstructions with fibula grafts, Hanasono *et al.* (14) concluded that CAD/CAM and stereolithographic models increased their accuracy and speed.

To reduce the ischaemia time of the fibula graft, the donor pedicle should not be dissected until the bone osteotomy has been performed (20). The stereolithographic model of the fibula itself will also facilitate the preparation of the fibula graft, resulting in a reduction of surgical time and the period of ischaemia.

In 2016, Jeong *et al.* (3) published a study in which the total time of surgery, using fibula surgical guides, was significantly lower in comparison to conventional surgeries, and no additional osteotomies or bone remodelling were required.

Also, Rustemeyer *et al.* (5) emphasized that the use of computer-aided surgical guides reduced the graft ischaemia time, although time savings were used as a preoperative in the case planning. Ischaemia times are critical for decreasing postoperative complications, as shown by Chang *et al.* (12). In their study of 116 patients undergoing mandibular reconstructions with osteomyocutaneous grafts of fibula, they concluded that the ischaemia should be less than five hours (13).

In our article, we describe two clinical cases of a diagnosed mandibular epidermoid carcinoma and a mandibular osteosarcoma in which surgical guides of free graft extraction, which were previously made by a stereolithography, were used and allowed to obtain enough graft to reconstruct the defects derived from tumor resection. In the third case, intraoperative navigation was also used to verify the limits of the planned tumor recession. We also used a stereolithographic model of the fibula with its arterial vascularization.

This article describes the main features of our experience in the area of mandibular reconstructions with software and 3-D printing with our own resources where we consider that the planning of the surgery is the key to obtaining good results in large facial reconstructions, basing the success of the surgical intervention on a greater precision of the mandibular reconstructions and in the reduction of the time of intervention, which is fundamental for a greater survival of the fibula graft.

The design, manufacture, and use of free fibula-graft surgical guides is essential for the success of these treatments. Among its advantages, besides the saving of operative and postoperative times, we must emphasize the reliability and accuracy at the time of obtaining the graft in the donor zone and take it to the receiving area, which covers all the desired spaces in the reconstruction and coincides with the conclusions of previous publications.

The current literature shows limitations in this topic since most of the published articles present a limited series of cases. We believe that prospective protocolized studies should be continued in the future to evaluate a larger sample of patients and to obtain a greater control of the different variables that could be involved in the results of the treatment.
